# A Case of Bilateral Adrenal Hemorrhage: The Exceptional Cause of Adrenal Insufficiency

**DOI:** 10.7759/cureus.23413

**Published:** 2022-03-23

**Authors:** Aemen S Khakwani, Fatima Waqar, Usman A Khan, Muhammad Nadeem Anwar

**Affiliations:** 1 Internal Medicine, Suburban Community Hospital, Norristown, USA; 2 Internal Medicine, Jefferson Abington Hospital, Abington, USA; 3 Internal Medicine/Nephrology, University of Oklahoma Health Sciences Center, Oklahoma City, USA; 4 Internal Medicine, Oklahoma City Veterans Affairs (VA) Medical Center, Oklahoma City, USA

**Keywords:** hydrocortisone, serum cortisol, acth, primary adrenal insufficiency, bilateral adrenal haemorrhage

## Abstract

Bilateral adrenal hemorrhage is an extremely uncommon and life-threatening condition. It is caused by multiple etiologies, including antiphospholipid syndrome, disseminated histoplasmosis, trauma, severe stress, and granulomatous disease. The authors present a unique case of a 64-year-old alcoholic male, who was admitted after fall and right hip fracture. On day seven of admission, the patient started to develop hypotension, leukocytosis, and tachycardia. CT abdomen was done, which ruled out infectious causes, however, it showed bilateral adrenal hemorrhages. Patient adrenocorticotrophic hormone (ACTH) stimulation test was positive for adrenal insufficiency and was started on hydrocortisone replacement. Our case highlights the fact that adrenal insufficiency after bilateral adrenal hemorrhage can be slow and can manifest as late as seven days and prompt therapy with steroids is warranted to avoid life-threatening adrenal insufficiency.

## Introduction

Bilateral adrenal hemorrhage is a rare endocrinologic emergency that carries a mortality rate of 15% [1,2]. Most cases are associated with severe hemodynamic compromise, sepsis, use of anticoagulants, antiphospholipid syndrome, disseminated histoplasmosis, trauma, severe stress, and granulomatous disease [3]. We present a unique case of a 64-year-old alcoholic male, who was admitted after fall and right hip fracture. The patient underwent right hip hemiarthroplasty. His hospital course was complicated by post-op ileus. On day seven of hospitalization, the patient started to develop hypotension, leukocytosis, and tachycardia. CT abdomen was done which ruled out infectious causes, however, it showed bilateral adrenal hemorrhages. The patient's adrenocorticotrophic hormone (ACTH) stimulation test was positive for adrenal insufficiency and was started on hydrocortisone replacement. The case highlights the fact that adrenal insufficiency after bilateral adrenal hemorrhage can be slow and can manifest as late as seven days and prompt therapy with steroids is warranted to avoid life-threatening adrenal insufficiency.

## Case presentation

**Figure 1:**
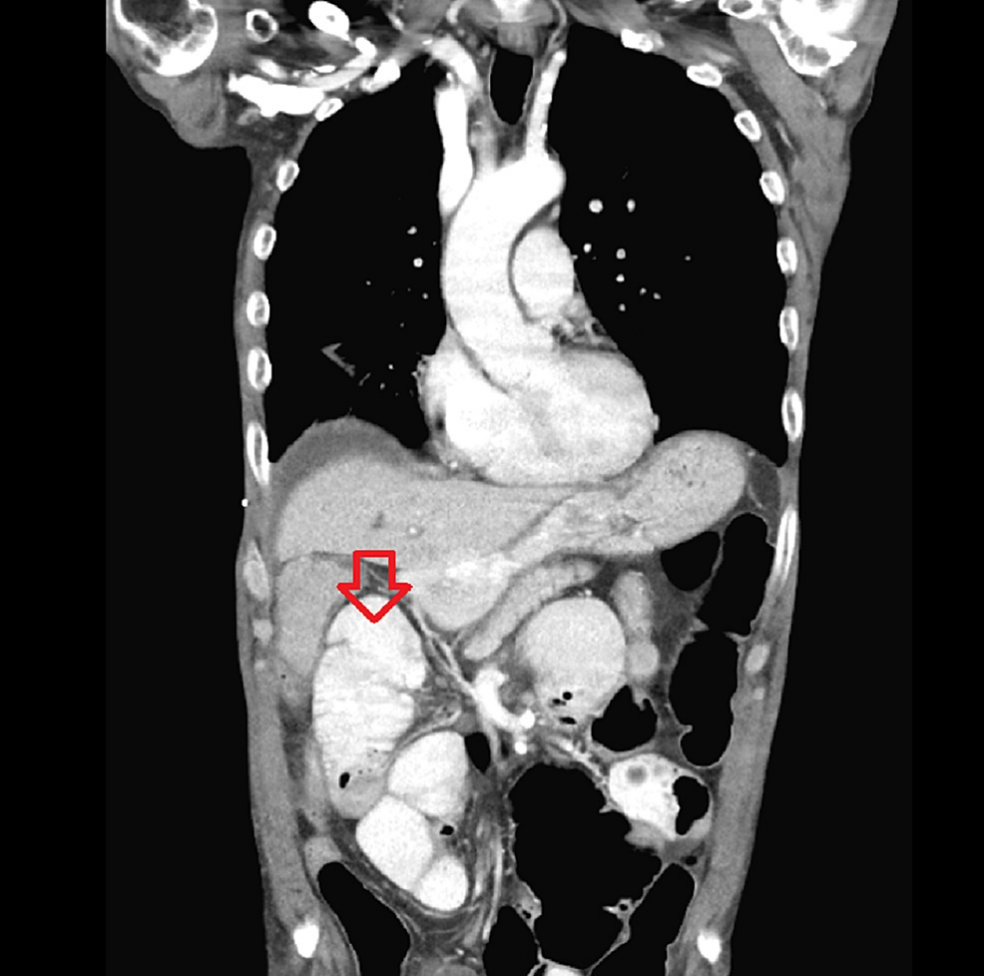
New hypoattenuation, enlargement, and stranding of the right adrenal gland showing acute-to-subacute hemorrhage

**Figure 2:**
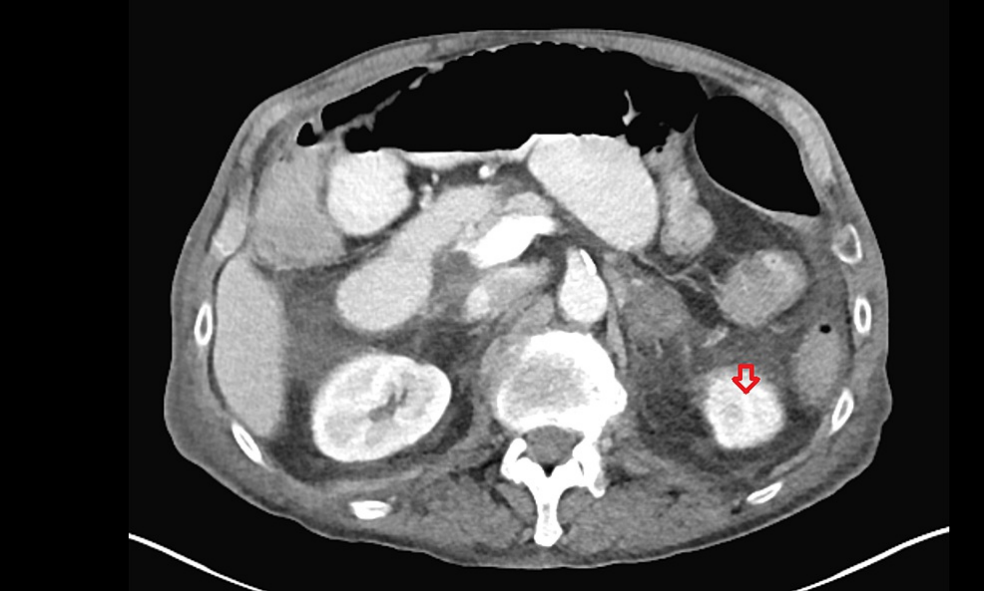
New hypoattenuation, enlargement, and stranding of the left adrenal gland showing acute-to-subacute hemorrhage

A 64-year-old white man was admitted to the hospital because of fall and right hip fracture. The patient had significant past medical history of alcohol use, bladder cancer status post cystectomy, urostomy, and hypertension. The patient underwent right hip hemiarthroplasty; postoperative course was complicated by paralytic ileus which improved with conservative care. On day seven of hospitalization, the patient started complaining of vague abdominal pain and fatigue. The patient started to get tachycardic in 120s beats per minute, blood pressure was 94/60 mmHg. Baseline labs showed leukocytosis of 14,000 k/cmm. CT abdomen was done to rule out any intra-abdominal infection and any possible source of sepsis. CT abdomen was reported as new enlargement and hypoattenuation of the adrenal glands, left greater than right with surrounding stranding suggesting an acute to subacute hemorrhages, which was not present on a CT abdomen done for paralytic ileus five days ago (Figures 1, 2).

Workup for bilateral adrenal hemorrhage including antiphospholipid antibodies, urine histoplasma antigen, and blood cultures was sent and reported to be negative. Cosyntropin stimulation test was done, which showed cortisol levels of 12 nmol/L at baseline, 11 nmol/L at 30 minutes, and 10 nmol/L at 60 minutes. The patient was started on hydrocortisone 20 mg in the a.m. and 10 mg in the p.m. after endocrinology consultation. The patient's blood pressure and tachycardia markedly improved and his leukocytosis resolved, and he was discharged with an outpatient endocrinology follow-up.

## Discussion

Adrenal hemorrhage is an uncommon condition and is caused by several etiologies including granulomatous infections, amyloidosis, septic shock, infiltrative diseases (lymphoma/leukemia), and adrenal carcinomas [4]. Ketha et al. reported a case series of four patients with bilateral adrenal hemorrhages secondary to heparin-induced thrombocytopenia, the underlying cause of adrenal hemorrhage has yet to be fully elucidated [5]. Some suggest reduced capillary resistance as a result of aging may be a factor [6], other's hypothesized that elevated stress hormones and ACTH as a result of stress produces severe vasoconstriction and platelet aggregation and lead to reperfusion and subsequent bleeding, particularly in the capillaries within the distal corticomedullary junction [7].

Adrenal insufficiency usually presents with acute onset of fever, nausea, vomiting, weakness, dizziness, fatigue, and epigastric pain. Our patient did not show any signs or symptoms of adrenal insufficiency until the seventh day into the hospital course when he started to have abdominal pain, fatigue, tachycardia, and hypotension. Importantly, our case raises the association of abdominal pain to adrenal hemorrhage, which has been previously described as one of the alarming signs of bilateral adrenal hemorrhage in literature.

The standard diagnostic assessment of adrenal hemorrhage includes CT with or without the use of contrast which shows hypoattenuation/densities of the adrenal glands. On review of literature, bilateral adrenal hemorrhage has a variable duration of presentation of adrenal insufficiency; experts say it may range from days to weeks before symptoms can manifest. Our patient failed the cosyntropin stimulation test, and his cortisol levels were recorded as 12 nmol/L, 11 nmol/L, and 10 nmol/L at baseline, 30, and 60 minutes, respectively. Despite late manifestation of signs and symptoms of adrenal insufficiency in our patient, we promptly started him on hydrocortisone 20 mg in the a.m. and 10 mg in the p.m. to avoid adrenal crisis, which resulted in marked clinical improvement emphasizing the fact that timely mineralocorticoid/glucocorticoids replacement is associated with improved morbidity and mortality.

## Conclusions

In summary, we presented a case of a 64-year-old male with bilateral adrenal hemorrhage most likely secondary to trauma and stress response. The patient only showed vague symptoms of abdominal pain and hypotension late in the hospitalization course; we wanted to alert the physicians to consider bilateral adrenal hemorrhage as one of the possibilities in patients who develop abdominal pain and hypotension in severely sick, septic, and trauma patients to identify adrenal hemorrhages and avoid adrenal crisis. The case highlights the fact that adrenal insufficiency after bilateral adrenal hemorrhage can be slow and can manifest as late as seven days and prompt therapy with steroids is warranted to avoid life-threatening adrenal insufficiency.
